# Mesh Inguinal Hernia Repair and Appendectomy in the Treatment of Amyand's Hernia with Non-Inflamed Appendices

**DOI:** 10.1155/2017/7696385

**Published:** 2017-01-17

**Authors:** Emin Kose, Abdullah Sisik, Mustafa Hasbahceci

**Affiliations:** ^1^Okmeydani Education and Research Hospital, General Surgery Clinic, Darulaceze Str. No. 25, Sisli, Istanbul, Turkey; ^2^Umraniye Education and Research Hospital, General Surgery Clinic, Adem Yavuz Str No. 1, Umraniye, Istanbul, Turkey; ^3^Department of General Surgery, Faculty of Medicine, Bezmialem Vakif University, Vatan Str., Fatih, 34093 Istanbul, Turkey

## Abstract

Amyand's hernia is defined as protrusion of the vermiform appendix in an inguinal hernia sac. It is a rare entity with variable clinical presentation from normal vermiform appendix to abscess formation due to perforation of acute appendicitis. Although surgical treatment includes appendectomy and hernia repair, appendectomy in the absence of an inflamed appendix and use of a mesh in cases of appendectomy remain to be controversial. The aim of this study was to review the experience of mesh inguinal hernia repair plus appendectomy performed for Amyand's hernia with noninflamed appendices. There were five male patients with a mean age of 42.4 ± 16.1 years in this retrospective study in which Amyand's hernia was treated with mesh inguinal hernia repair plus appendectomy for noninflamed appendices. Patients with acute appendicitis and perforated vermiform appendix were excluded. There were four right sided and one bilateral inguinal hernia. Postoperative courses were uneventful. During the follow-up period (14.0 ± 7.7 months), there was no inguinal hernia recurrence. Mesh inguinal hernia repair with appendectomy can be performed for Amyand's hernia in the absence of acute appendicitis. However, presence of fibrous connections between the vermiform appendix and the surrounding hernia sac may be regarded as a parameter to perform appendectomy.

## 1. Introduction

Amyand's hernia (AH) is defined as protrusion of the vermiform appendix in an inguinal hernia sac [1]. This entity was named historically by Amyand at 1736 [2]. In almost 1% of all inguinal hernias, AH is detected and acute appendicitis in AH cases accounts only for 0.1% [1]. Thus, AH is a rare entity with variable clinical presentation from normal vermiform appendix to abscess formation due to perforation of acute appendicitis.

It is generally accepted that surgical treatment of AH includes both appendectomy and hernia repair [1, 3, 4]. However, appendectomy in the absence of an inflamed appendix and use of a mesh in cases of appendectomy remain to be controversial. Some authors offer not to perform prophylactic appendectomy when noninflamed appendix is incidentally found in the hernia sac [3]. However, others believe that appendectomy should be performed in all cases to prevent future reherniations and appendicitis [5, 6].

Although mesh repair has been used even in perforated or mildly inflamed cases of AH, it is generally accepted that hernia repair with mesh should be avoided in cases of appendectomy performed for noninflamed or inflamed appendices [3, 5, 7–13]. Additionally, there have been some classification systems with regard to its presentation and treatment recommendations [1, 14, 15]. According to these findings, controversies with regard to features of AH and treatment options are still present because no evidence-based information exists [7].

It has been thought that it is impossible to reach sufficient number of AH cases to get evidence-based data due to its rarity. Therefore, it is logical to revise the classification and surgical treatment of AH based on the case reports from different institutions.

The aim of this study was to review the experience of mesh inguinal hernia repair plus appendectomy performed for AH with noninflamed appendices.

## 2. Material and Methods

This study was a retrospective analysis of the patients with AH treated via appendectomy with mesh inguinal hernia repair. Written consent was taken from the patients and the approval was taken from the institutional review board.

The diagnosis of AH was performed intraoperatively by inspecting normal appearance of the vermiform appendix in the inguinal hernia sac during the hernia repair. The cases with AH were classified into three types according to the system proposed by Fernando and Ceulemans [14, 15]. Types A and B represented AH with intact vermiform appendix without and with signs of inflammation, respectively. Type C was used for AH with perforated vermiform appendix. Therefore, only the cases with type A were included in the study. Patients with acute appendicitis (type B), perforated vermiform appendix (type C), and other intra-abdominal pathologies including gastrointestinal malignancies were excluded. Surgical treatment was performed by the authors.

Demographic data including age, sex, intraoperative findings, type of surgery, and outcome were collected from the patients' records.

## 3. Surgical Technique

**Figure 1:**
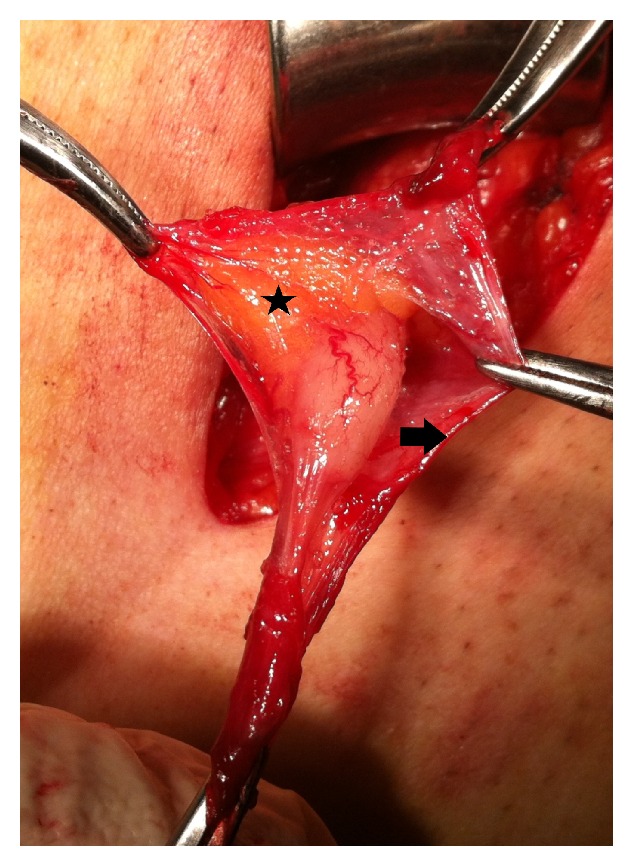
Normal appearing the vermiform appendix in the inguinal hernia sac. Fibrous connections (star) between the vermiform appendix and surrounding hernia sac (arrow).

**Figure 2:**
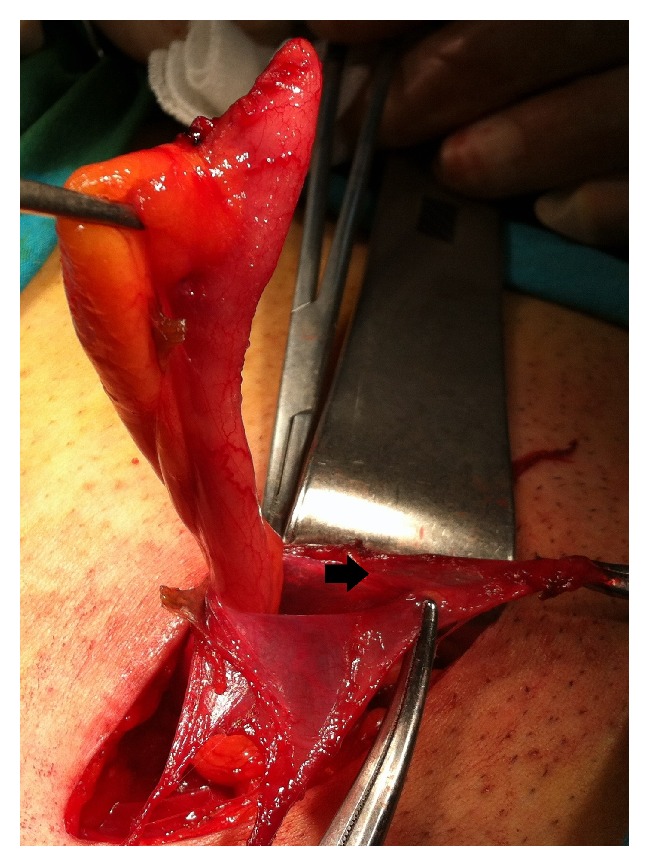
After the dissection, the vermiform appendix located within the inguinal hernia sac (arrow).

**Figure 3:**
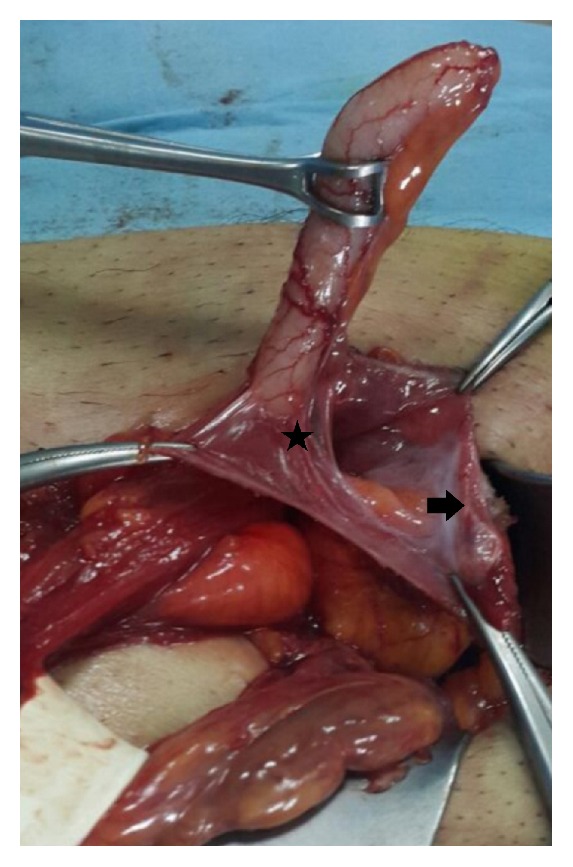
Normal appearance of the vermiform appendix in the inguinal hernia sac. Severe adhesions (star) between the vermiform appendix and surrounding hernia sac (arrow).

**Figure 4:**
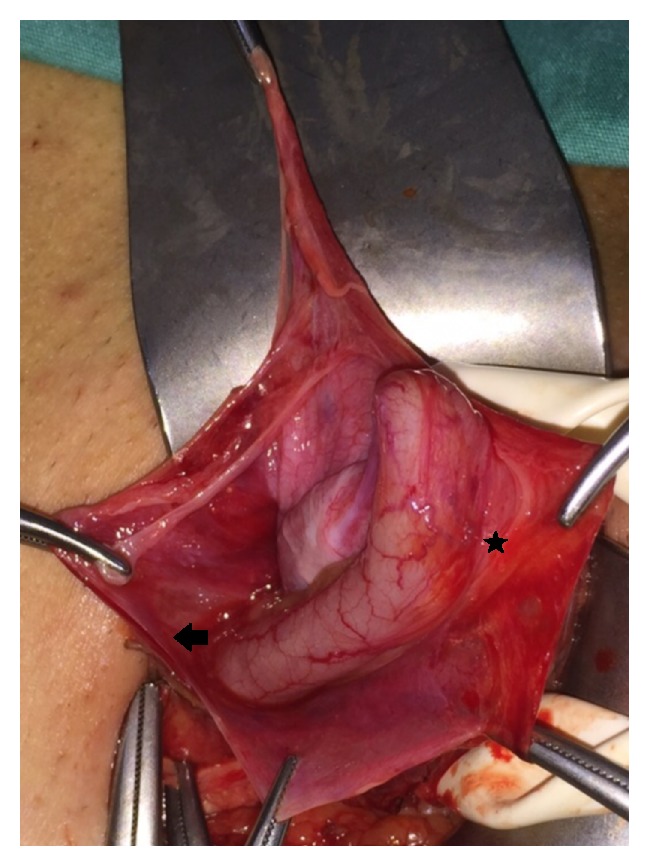
Minimally inflamed vermiform appendix in the inguinal hernia sac. Severe adhesions (star) between the vermiform appendix and surrounding hernia sac (arrow).

All of the cases were done under general or regional anesthesia. Preoperative prophylactic antibiotic was given to all as sefazolin sodium 1 gr intravenously. An inguinal incision over the hernia was performed. During dissection of the hernia sac, a tubular structure corresponding to the vermiform appendix was discovered (Figures 1–4). The vermiform appendix could be freed from the hernia sac with dissection. Under strict protection of the surgical borders, appendectomy was performed via the inguinal incision. Then, the hernia sac was closed. Mesh hernia repair was performed as described by Amid et al. [16].

## 4. Results

There were five male patients in the study group with a mean age of 42.4 ± 16.1 years. There were four right sided and one bilateral inguinal hernia. Appendectomy plus mesh hernia repair was performed in all. Postoperative courses were uneventful. During the follow-up period (14.0 ± 7.7 months), there was no inguinal hernia recurrence.

## 5. Discussion

Surgical treatment of AH has been regarded as a challenging issue according to the reports published before varying from tissue repair of inguinal hernia to the hernia repair with biological meshes with or without appendectomy. Although presence of acute appendicitis or associated complications necessitates appendectomy in patients with AH, the type of inguinal hernia repair is getting more important. Additionally, it is believed that salient features of the vermiform appendix detailed in previous classification systems (Table 1) have some missing points including presence of fibrous connections between the vermiform appendix and the hernia sac that could not be freed without dissection [1, 14, 15]. Therefore, surgical treatment of AH should be revised with regard to these controversial issues even if it is a rare pathology.

Type 1 or type A AH is defined as the inguinal hernia with normal vermiform appendix within the sac according to Losanoff and Basson, Fernando and Ceulemans [1, 7, 14, 15] (Table 1). In literature, there has been a great agreement on the surgical treatment of AH with complicated appendicitis (type 3, type 4, or type C) [17]. However, controversies have been reported for other types (type 1, type 2 or type A, and type B). For type 1 or type A AH, mesh inguinal hernia repair has been recommended with the benefits of an improved longevity of the repair if appendectomy was not performed [7]. However, the decision to remove or leave behind a normal vermiform appendix in type 1 or type A AH has not been determined yet [1, 3, 7]. Some authors have thought that appendectomy should be performed only if the appendix is inflamed or perforated [1]. Although age, life expectancy, or lifelong risk of having acute appendicitis is the parameter that can be considered, our opinion based on the surgeon's personal choice was to remove the normally appearing appendix to prevent future complications while taking the patients' characteristics into consideration in accordance with other authors [12, 18, 19].

There has been a general tendency as not to use mesh for hernia repair in cases with appendectomy performed for noninflamed appendices [1, 3, 7, 20]. However, use of mesh in these circumstances and additionally in cases with appendectomy for inflamed appendices (type 2 or type B) has been reported by many researchers [8, 17, 21]. Interestingly, complications due to mesh usage in surgical treatment of AH have not been reported in these papers as in the present study. In some studies, endogenous tissues or use of biological mesh such as acellular collagen materials has been recommended for type 2 or type B AH albeit at the risk of an increased cost and recurrence [7, 22]. Therefore, mesh inguinal hernia repair can be regarded as a safe technique in combination with appendectomy performed for both noninflamed and inflamed appendices [10, 13, 17].

Fibrous connections between the vermiform appendix and surrounding hernia sac preventing free reduction of the vermiform appendix to the abdominal cavity were thought to be an important clinical parameter to decide appendectomy based on the surgical experience gathered from the cases in this study. Manipulation and surgical maneuvers to dissect the appendix may cause more inflammation provoking secondary appendicitis and adhesion just below the parietal peritoneum even if it is noninflamed [18, 23]. Although evidence-based data with regard to this issue cannot be produced due to the ethical problems, our approach was to remove the normal appendix in cases of AH.

The main limitation of the study was the short number of the patients with the short follow-up period. Although this may cause underestimation of the exact incidence of complications due to the use of mesh and recurrences, it is logical to expect to see the complications due to the use of mesh within almost one year. However, more studies including more patients with longer follow-up period are needed to clarify this problem.

## 6. Conclusion

Mesh inguinal hernia repair with appendectomy can be performed for the surgical treatment of AH type A considering the patients' characteristics rather than the therapeutic frame sets defined previously. Presence of fibrous connections between the vermiform appendix and the surrounding hernia sac may also be regarded as a parameter to perform appendectomy in surgical treatment of type A AH.

## Figures and Tables

**Table 1:** Classification systems for Amyand's hernia.

Types	Type 1^**∗**^/A^†^	Type 2/B	Type 3/C	Type 4
Salient feature	Normal appendix/noninflamed	Acute appendicitis localized in the sac/inflamed	Acute appendicitis, peritonitis/perforated	Acute appendicitis, other abdominal pathology
Surgical management^**∗**^	Reduction or appendectomy (depending on age), mesh hernioplasty	Appendectomy through hernia, endogenous repair	Appendectomy through laparotomy, endogenous repair	Appendectomy, diagnostic workup, and other procedures as appropriate

^**∗**^Types from 1 to 4 (Losanoff-Basson)

^†^Types from A to C (Fernando and Ceulemans).
